# Low Double-Negative CD3^+^CD4^−^CD8^−^ T Cells Are Associated with Incomplete Restoration of CD4^+^ T Cells and Higher Immune Activation in HIV-1 Immunological Non-Responders

**DOI:** 10.3389/fimmu.2016.00579

**Published:** 2016-12-09

**Authors:** Xiaofan Lu, Bin Su, Huan Xia, Xin Zhang, Zhiying Liu, Yunxia Ji, Zixuan Yang, Lili Dai, Luzia M. Mayr, Christiane Moog, Hao Wu, Xiaojie Huang, Tong Zhang

**Affiliations:** ^1^STD/HIV Research Laboratory, Beijing You’an Hospital, Capital Medical University, Beijing, China; ^2^Beijing Key Laboratory for HIV/AIDS Research, Beijing, China; ^3^Center for Infectious Diseases, Beijing You’an Hospital, Capital Medical University, Beijing, China; ^4^INSERM UMR S_1109, Faculté de Médecine, FMTS, Centre de Recherche en Immunologie et Hématologie, Université de Strasbourg, Strasbourg, France

**Keywords:** HIV-1, double-negative T cell, immune reconstitution, immune activation, antiretroviral therapy, TGF-β1

## Abstract

Failure of immune reconstitution increases the risk of AIDS or non-AIDS related morbidity and mortality in HIV-1-infected patients. CD3^+^CD4^−^CD8^−^ T cells, which are usually described as double-negative (DN) T cells, display CD4-like helper and immunoregulatory functions. Here, we have measured the percentage of DN T cells in the immune reconstituted vs. non-immune reconstituted HIV-1-infected individuals. We observed that immunological non-responders (INRs) had a low number of DN T cells after long-term antiretroviral therapy (ART), and the number of these cells positively correlated with the CD4^+^ T cell count. The ART did not result in complete suppression of immune activation recorded by the percentage of CD38^+^HLA-DR^+^CD8^+^ T cells in INRs, and a strong inverse correlation was observed between DN T cells and immune activation. A low proportion of TGF-β1^+^DN T cells was found in INRs. Further mechanism study demonstrated that the level of TGF-β1-producing DN T cells and immune activation had a negative correlation after ART. Taken together, our study suggests that DN T cells control the immunological response in HIV-1-infected patients. These findings expand our understanding of the mechanism of immune reconstitution and could develop specific treatments to return the immune system to homeostasis following initiation of HIV-1 therapy.

## Introduction

CD3^+^CD4^−^CD8^−^ double-negative (DN) T cells constitute approximately 1–5% of T lymphocytes in mice, non-human primates, and humans (1–4). These cells are also found in the lymph nodes, lungs, and gut-associated lymphoid tissues (5–8). They predominately have the memory phenotype (9), and it has been suggested that they play an important function in graft-vs.-host disease (10), autoimmune diseases (11), parasite infection (12), and infectious disease, such as HIV/SIV infection (5, 9, 13–15). DN T cells not only have a regulatory function similar to regulatory T cells (Tregs) but also secrete CD4-like cytokines (IL-4, IL-17, IFN-γ, and TNF-α) to exert their T helper function (4, 5, 12, 16, 17). In SIV-infected natural hosts, such as sooty mangabeys and African green monkeys, the frequency of DN T cells is about 10–40% of the total circulating lymphocytes (4). The maintenance of high numbers of these DN T cells capable of secreting T helper cytokines is associated with immune activation, and thus it is proposed that this can contribute to the prevention of SIV disease progression in natural host monkey species (4, 5, 9). In primary HIV-1-infected individuals, elevated DN T cells are associated with the control of immune activation and lower viral load (14). In addition to secreting IL-10 and TGF-β cytokine (14), DN T cells are also involved in restraining the CD8^+^ T cell immune activation during primary HIV-1 infection (14). During the chronic phase, a decrease of DN T cells is correlated with rapid disease progression (13). Moreover, recently, DN T cells were identified as the latent HIV-1 reservoir that harbors HIV-1 Gag protein in the presence of antiretroviral therapy (ART) (18).

Nearly 20–30% of the HIV-1-infected patients lack the ability to fully recover CD4^+^ T cells after long-term ART (19, 20). These patients are considered as immunological non-responders (INRs). The failure of immune reconstitution eventually leads to the progression of AIDS or non-AIDS events, thereby increasing the morbidity and mortality in HIV/AIDS patients (21, 22). The underlying causes associated with INRs are age, low nadir CD4, high immune activation, and impaired thymus function (23–25).

The role of DN T cells in immune reconstitution has not been investigated. In the present study, the distribution of DN T cells and CD38^+^HLA-DR^+^CD8^+^ T cells as marker of immune activation was assessed in immunological responders (IRs) vs. INRs after prolonged ART in HIV-1 chronically infected patients. We found a higher number of DN T cells to be associated with a decreased number of CD38^+^HLA-DR^+^CD8^+^ T cells in IRs patients, suggesting that DN T cells play a role in immune restoration. Moreover, the production of TGF-β1 by DN T cells might participate in the downregulation of immune activation after long-term ART.

## Materials and Methods

### Study Subjects and Ethical Issues

Cryo-preserved peripheral blood mononuclear cells (PBMCs) were obtained from ART-treated chronic HIV-1-infected cohorts present in Beijing You’an Hospital. HIV-1 patients received ART for at least 1 year post-infection, and their CD4^+^ T cell count had dropped below 350 cells/μl. These patients were divided into two groups following a minimum of 2 years of ART: 17 IRs (CD4 > 500 cells/μl) and 19 immunological non-responders (INRs, CD4 < 350 cells/μl). Eighteen age-matched healthy controls (HCs) were also included in this study. All the participants provided written informed consent. This study and all relevant experiments have been approved by the Beijing You’an Hospital Research Ethics Committee. The methods were carried out in accordance with the approved guidelines and regulations.

### Immunophenotyping

Cryo-preserved PBMCs were thawed in RPMI 1640 medium (Invitrogen, Carlsbad, CA, USA); then, these cells were washed with PBS containing 1% BSA. Briefly, cells were incubated at room temperature for 20 min with the cell viability marker fixable viability stain 510 (BD Biosciences, San Diego, CA, USA) and the following monoclonal conjugated antibodies: CD3-PE-cy7 (SK7), CD4-APC-cy7 (SK3), CD8-PE (RPA-T8), CD8-percp-cy5.5 (RPA-T8), HLA-DR-APC (TU36), and CD38-PE (HIT2) (BD Biosciences). Cells were washed and fixed with 2% paraformaldehyde, and at least 50,000 live lymphocytes were acquired with a BD FACSCanto II Flow Cytometry Analyzer Systems (BD Biosciences). Forward angle and side scatter light gating were used to exclude cell debris from the analysis. Forward width and forward area were used to exclude doublet cells. The final analysis was performed using the Flowjo7.6.1 software (Tree Star Inc., Ashland, OR, USA).

### Intracellular Cytokine Staining

Peripheral blood mononuclear cells (1 × 10^6^) were stimulated with PMA (500 ng/ml), ionomycin (2 μg/ml), and brefeldin A (10 μg/ml) for 5 h at 37°C in 5% CO_2_. The surface were stained with CD3-BV421 (UCHT1), CD8-percp-cy5.5 (RPA-T8), and CD4-FITC (RPA-T4) (BD Biosciences), and intracellular staining was performed using anti-IL-17A-PE (N49-653), IL-10-APC (JES3-19F1) (BD Biosciences), IL-4-APC (8D4-8), TGF-β1-PE (TW4-6H10), TNF-α-PE-cy7 (MAb11), and IFN-γ-PE-cy7 (4S.B3) (Biolegend, San Diego, CA, USA) and the foxp3/transcription factor staining buffer set (eBioscience, San Diego, CA, USA) according to the manufacturer’s protocols. Unstimulated cells were used as a negative control.

### CD4^+^ T Cell Count and Viral Load Measurement

Routine blood CD4^+^ T cell counts (cells per microliter) were measured by four-color flow cytometry using human CD45^+^, CD3^+^, CD4^+^, and CD8^+^ cell markers (BD Biosciences) in whole peripheral blood samples from each patient using FACS lysing solution (BD Biosciences) according to the manufacturer’s instructions. Plasma HIV-1 viral load (copies per milliliter of plasma) was quantified by real-time PCR (Abbort, Des Plaines, IL, USA). The sensitivity of viral RNA detection using this assay is 40 copies/ml of plasma.

### Statistical Analysis

All statistical analyses were performed using the GraphPad Prism software v5.0 (GraphPad Software, Inc., La Jolla, CA, USA). The statistical significance between two groups was calculated using Mann–Whitney *U* test, and correlations were determined by the Spearman rank correlation test, with *r* being the Spearman correlation coefficient. The *p* < 0.05 was considered statistically significant.

## Results

### Characterization of the Subjects

Thirty-six HIV-1-infected patients who had sustained undetectable viral load after treatment were divided into two groups: IRs (*n* = 17) and INRs (*n* = 19) according to their CD4^+^ T cell level recorded after at least 2 years of treatment. Eighteen age-matched HCs were also part of this study (Table 1). These HIV-1-infected patients had no HBV or HCV co-infection. The plasma HIV-1 viral load between IR and INR groups did not show any statistically significant difference before ART. The frequencies of CD4^+^ T cells also did not differ significantly in these two groups of patients, before or after ART, respectively. However, CD4^+^ T cell count in IR group patients was higher than in INR group patients (*p* = 0.002 before ART; *p* < 0.0001 after ART) and reached the level of HCs after ART administration.

**Table 1:** **Characteristics of study subjects**.

		HIV-infected patients		*p* Value
	Healthy controls (*n* = 18)	Immunological responders (IRs) (*n* = 17)	Immunological non-responders (INRs) (*n* = 19)	IR vs. INR
Mean age (range)	38.13 (30–45)	38.76 (30–46)	37.12 (31–44)	ns
**Mean CD4% (range)**				
pre-ART	42 (21.9–67.2)	21.69 (9.51–53.9)	20.38 (6.4–40)	0.81
ART		31.07 (13.2–43.8)	31.08 (9.23–38.8)	0.47
**Mean CD4 count (cells/μl) (range)**				
pre-ART	770.27 (484–1,238)	255.52 (183–336)	100 (88–292)	0.002
ART		718.7 (608–925)	272.15 (154–326)	<0.0001
**Mean viral load (log10, copies/ml) (range)**				
pre-ART	NA	4.32 (3.14–5.74)	4.02 (1.97–5.07)	ns
ART		<40	<40	NA

*Data are presented as mean (range)*.

*p values were determined by Mann–Whitney U two-tailed t-test*.

*NA, not applicable; ns, not significant*.

### Long-term ART Only Partially Restored the Level of DN T Cells in the INR Group of Patients

**Figure 1:**
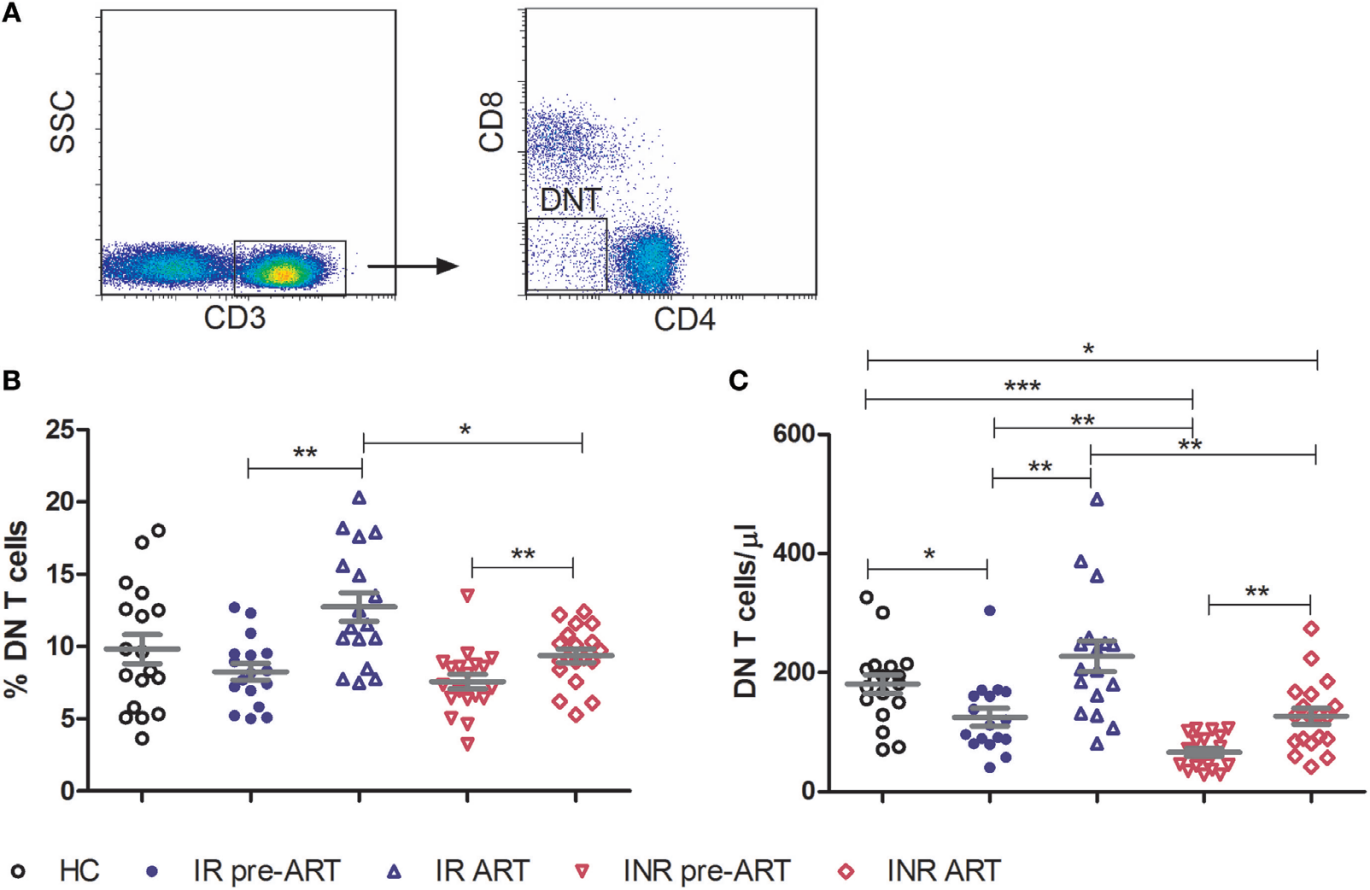
**Double-negative (DN) T cells are partially restored by prolonged antiretroviral therapy in immunological non-responders (INRs)**. **(A)** Representative flow cytometric plots of DN T cells from a healthy control (HC). Frequency **(B)** and count **(C)** of DN T cells were compared among three groups of study subjects (HC: *n* = 18, immunological responders: *n* = 17, and INR: *n* = 19). Mann–Whitney *U* test was used for statistical analysis. Horizontal lines indicate mean values, and error bars represent mean ± SEM (*p < 0.05, **p < 0.01, ***p < 0.0001).

**Figure 2:**
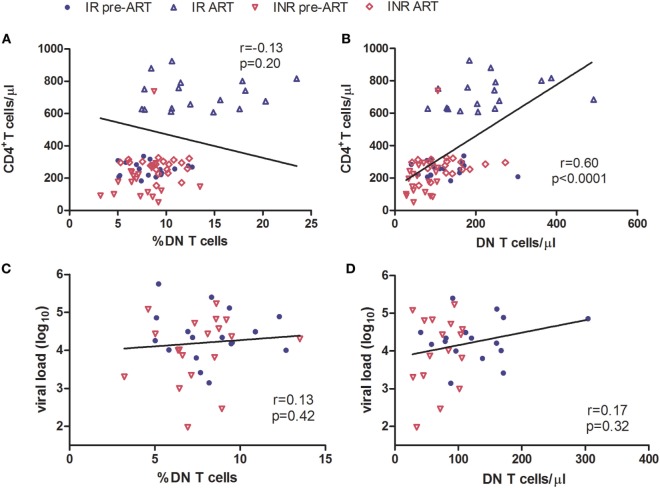
**Correlation of double-negative (DN) T cells with CD4^+^ T cell count**. Correlation of the frequency **(A)** and count **(B)** of DN T cells with CD4^+^ T cell count, among HIV-1-infected patients before and after long-term antiretroviral therapy (ART). Correlation of the frequency **(C)** and count **(D)** of DN T cells with viral load among study subjects, before ART. The Spearman rank test was used for correlation analysis.

Double-negative T cells were defined as CD3^+^ T cells that lack CD4 and CD8 expression (Figure 1A). Consistent with previous published studies, chronic HIV-1 infection significantly reduced the count of DN T cells in comparison to HCs. Interestingly, ART was able to partially restore the number and percentage of DN T cells in all HIV-1-infected patients (Figure S1 in Supplementary Material). To further delineate the potential role of DN T cells in immune reconstitution, we analyzed their proportion and count according to their immune reconstitution status. As shown in Figures 1B,C, compared to HCs, the frequency of DN T cells in both IR and INR group was reduced with chronic HIV-1 infection, although this trend was not statistically significant. DN T cell count was significantly reduced in these two group patients (HC vs. IR, *p* < 0.05; HC vs. INR, *p* < 0.0001). However, the ART effectively restored the level of DN T cells in IR group toward that found in HCs. In contrast, the frequency and count of DN T cells in INR group were only partially recovered after prolonged ART and could not match the levels observed in HCs or IRs (mean frequency: IR vs. INR, *p* < 0.05; mean count: HC vs. INR, *p* < 0.05; IR vs. INR, *p* < 0.01). Of note, the correlation analysis revealed that the DN T cell count positively correlated with the total CD4^+^ T cell count (Figures 2A,B) but not with viral load (Figures 2C,D).

### INRs with Poor DN T Cell Restoration after ART Display Higher Immune Activation

**Figure 3:**
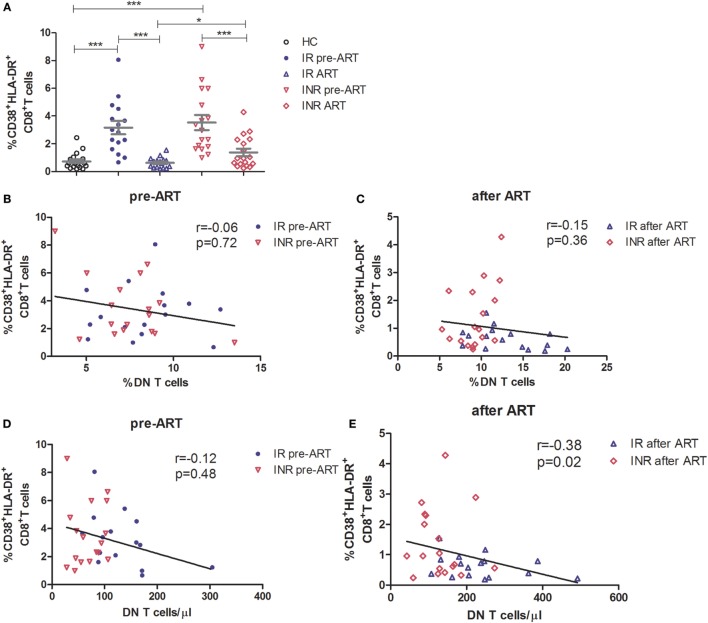
**High level of immune activation is associated with incomplete recovery of double-negative (DN) T cells after antiretroviral therapy (ART) in immunological non-responders**. **(A)** Co-expression of CD38 and HLA-DR on the surface of CD8^+^ T cells was compared among three groups of subjects. Mann–Whitney *U* test was used for statistical analysis. Horizontal lines indicate mean values, and error bars represent mean ± SEM (*p < 0.05, ***p < 0.0001). Relationship analysis between the frequency of DN T cells and the level of immune activation pre-ART **(B)** and after ART **(C)** or the DN T cell count and the level of immune activation pre-ART **(D)** and after ART **(E)**. The Spearman rank test was used for correlation analysis.

The regulatory function of DN T cells is responsible for limiting the immune activation in HIV/SIV infection (5, 14). Therefore, we next determined the relationship between DN T cells and the level of immune activation in immune reconstitution. Co-expression of CD38 and HLA-DR on the surface of CD8^+^ T cells was used as a marker to evaluate the degree of immune activation. Compared to HCs, both IRs and INRs showed a significant increased level of CD38^+^HLA-DR^+^CD8^+^ T cells during chronic HIV-1 infection (*p* < 0.0001) (Figure 3A). Although the long-term ART effectively reduced the overall immune activation among patients from both groups, the INR group displayed a higher level of CD38 and HLA-DR expression on CD8^+^ T cells in comparison to IRs (*p* < 0.05, Figure 3A). We next analyzed the relationship between immune activation and DN T cells in patients pre- or post-ART, respectively. We observed a significant negative correlation between the count of DN T cells and the level of CD38^+^HLA-DR^+^CD8^+^ T cells in patients after long-term ART (Figures 3B–E).

### TGF-β1 Produced by DN T Cells Participates in the Control of Immune Activation in HIV-1 Patients

**Figure 4:**
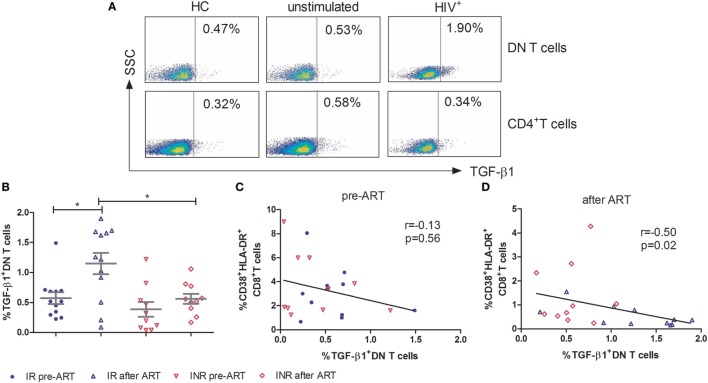
**Secretion of TGF-β1 by double-negative (DN) T cells contributes to the control of immune activation**. Peripheral blood mononuclear cells were cultured in medium only (unstimulated) or in the presence of PMA/Ionomycin for 5 h. Cytokine release was measured in DN T cells and CD4^+^ T cells by ICS. **(A)** Gating strategy for TGF-β1 in DN T cells or CD4^+^ T cells. **(B)** TGF-β1 expression on DN T cells was compared between immunological responders (*n* = 12) and immunological non-responders (*n* = 10). Mann–Whitney *U* test was used for statistical analysis. Horizontal lines indicate mean values, and error bars represent mean ± SEM (*p < 0.05). Correlation between TGF-β1 expressing DN T cells and the level of immune activation pre-antiretroviral therapy (ART) **(C)** and after ART **(D)**.

To further elucidate the mechanism of DN T cells in the control of immune activation, PBMCs were stimulated for 5 h with PMA/ionomycin and assessed for immunosuppressive (IL-10 and TGF-β1), Th1 (IFN-γ), Th2 (IL-4), Th17 (IL-17A), and pro-inflammatory (TNF-α) cytokine producing by DN T cells. As shown in Figure 4A, in contrast to CD4^+^ T cells, the TGF-β1 expressing DN T cells were detected in HIV-1 patients. In HCs, the expression of TGF-β1 by DN T cells was similar to that of unstimulated cells. The production of TGF-β1 in DN T cells was significantly unregulated after ART in IRs (*p* < 0.05); however, this increasing trend was not observed in INRs (Figure 4B). Furthermore, the level of TGF-β1-secreting DN T cells in IRs was much higher than that in INRs after ART (*p* < 0.05) (Figure 4B). Correlation analysis revealed that the proportion of TGF-β1 in DN T cells was negatively correlated with the level of immune activation after prolonged ART but not pre-ART in HIV-1 patients (Figures 4C,D). However, cytokines of IL-4, IL-10, IL-17A, IFN-γ, and TNF-α from DN T cells did not have different significance between IRs and INRs after ART (data not shown). Therefore, we proposed that low TGF-β1-producing DN T cells may contribute to the incomplete reduction of immune activation in INRs despite ART.

## Discussion

This study was performed to investigate the role of DN T cells in immune reconstitution of HIV-1-infected individuals. In INRs, both the frequency and count of DN T cells were lower than in IRs after long-term ART. In addition, INRs displayed higher levels of immune activation than IRs. Mechanism study demonstrated that the secretion of TGF-β1 by DN T cells may be involved in the control of immune activation.

Several studies have demonstrated that a high level of DN T cells is associated with a non-pathogenic outcome in SIV host species (4, 5, 9). Consistent with the SIV study, we have previously observed a decrease in the number of DN T cells in CD4^low^ HIV-1 patients (13). The INRs typically have a higher risk of opportunistic infections like AIDS or non-AIDS events than IRs and display increased morbidity and mortality (22). INRs and IRs have similar CD4^+^ T cell counts but the abnormal CD4^+^ T cell function may influence the outcome of antiviral immune responses (26). The DN T cells may compensate for the CD4^+^ T cells, therefore, poor restoration of these DN T cells in INRs (Figures 1B,C) may result in impaired immune responses during HIV-1 infection and thus accelerate the disease progression among these patients despite the fact that they have similar CD4^+^ T cell counts. Furthermore, the size and position of latent HIV-1 infection resulted in different progression in HIV-1 patients (27–29). HIV-1 latent reservoir was identified in DN T cells (18), therefore, we proposed that provirus harbored in DN T cells also contributes to the immune reconstitution failure in INRs.

The regulatory role of DN T cells has been demonstrated in HIV/SIV infection (5, 14). In SIV-infected sooty mangabeys, a lack of disease progression correlates with significant CD4^+^ T cell loss and is associated with DN T cells (5). These mangabeys exhibited low levels of immune activation, and the presence of DN T cells was believed to be responsible for this control (5). In primary HIV-1-infected individuals, elevated DN T cells were associated with the control of immune activation and lower viral load (14). The production of IL-10 and TGF-β1 by DN T cells has been suggested to participate to this outcome, which further supports the regulatory role of DN T cells (14). Consistent with these studies, we confirmed that INRs had higher immune activation than IRs after long-term ART, although ART resulted in an overall reduction in the immune activation as shown in Figure 3A. Moreover, we observed a negative correlation between DN T cells and the level of immune activation in HIV-1 patients after ART (Figure 3E). A high level of immune activation was previously proposed to contribute to the failure of immune reconstitution (23, 24). Further analysis revealed that the secretion of TGF-β1 by DN T cells was responsible for the control of immune activation after long-term ART (Figure 4). As we found that DN T cells were increased in IR patients, our results put forward the potential contribution of DN T cells in the process of immune reconstitution in HIV-1-infected patients.

In conclusion, our study demonstrated that INRs have lower levels of DN T cells and exhibit higher immune activation than IRs after long-term ART. Because DN T cells have both CD4-like and regulatory functions, the DN T cells in IRs may result in partial compensation of CD4^+^ T cell loss and in the control of immune activation, thereby improving the immunological response to ART in IRs.

## Author Contributions

XL, BS, XH, and TZ conceived the study, designed the experiments, and analyzed the data. XL, HX, XZ, ZL, YJ, and ZY performed the experiments; LD, XH, LM, and TZ contributed to reagents and materials; and XL, BS, CM, XH, and TZ wrote the article. All authors read and approved the final manuscript.

## Conflict of Interest Statement

The authors declare that the research was conducted in the absence of any commercial or financial relationships that could be construed as a potential conflict of interest. The reviewer MR declared a shared affiliation, though no other collaboration, with two of the authors LM and CM to the handling editor, who ensured that the process nevertheless met the standards of a fair and objective review.

## Supplementary Material

Figure S1 **Recovery of double-negative (DN) T cells in HIV-infected patients after long-term antiretroviral therapy (ART)**. Healthy control (*n* = 18) and chronic HIV-infected patients (*n* = 36) were recruited in this study. Fresh blood of patients was collected before and after ART; peripheral blood mononuclear cells were isolated and stored at liquid nitrogen. Flow cytometry was performed as described in methods and materials. Frequency **(A)** and count **(B)** of DN T cells are compared among three groups. Mann–Whitney *U* test was used for statistical analysis. Horizontal lines indicate mean values, and error bars represent mean ± SEM (****p* < 0.0001).
