# Supplementary figures and images for: Low Double-Negative CD3+CD4−CD8− T Cells Are Associated with Incomplete Restoration of CD4+ T Cells and Higher Immune Activation in HIV-1 Immunological Non-Responders
Source: Front Immunol. 2016 Dec 9;7:579. doi: 10.3389/fimmu.2016.00579 (PMC5145861; doi:10.3389/fimmu.2016.00579)

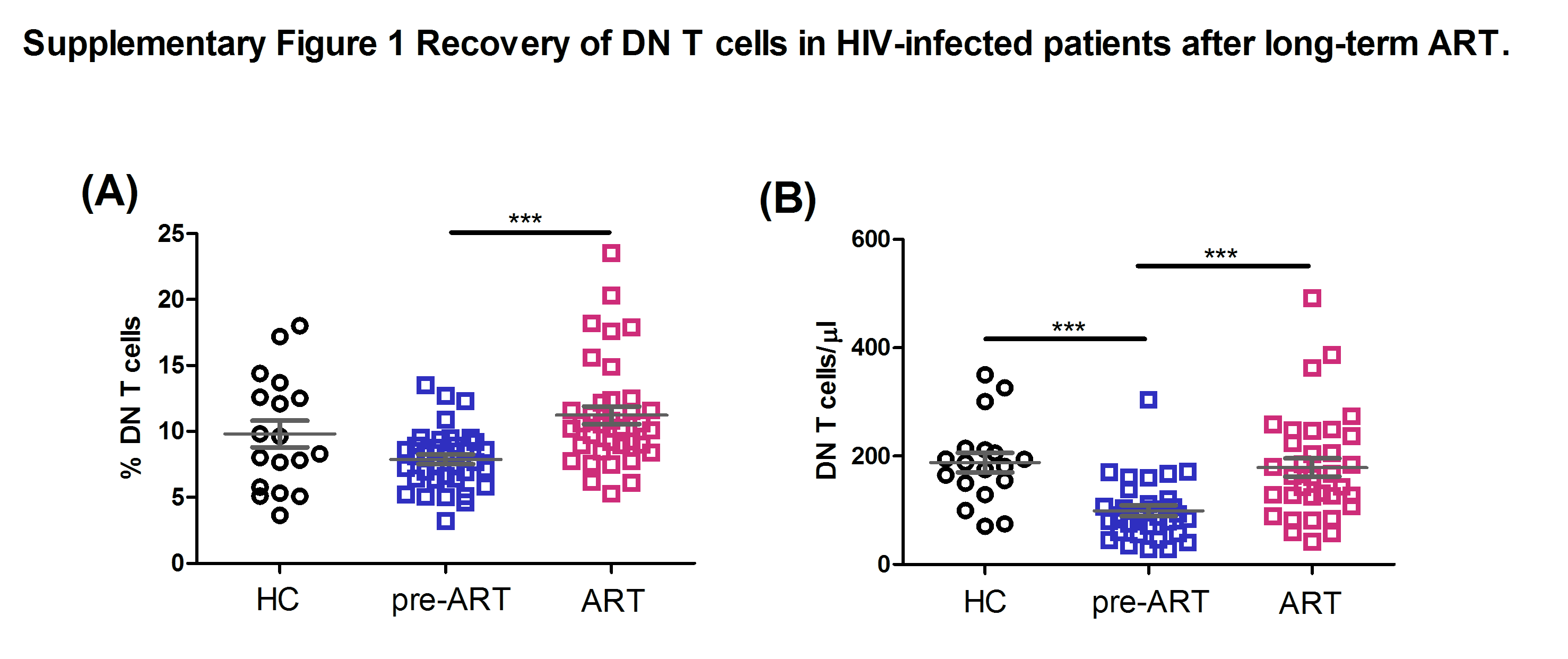

Supplement: Figure S1 — Recovery of double-negative (DN) T cells in HIV-infected patients after long-term antiretroviral therapy (ART). Healthy control (n = 18) and chronic HIV-infected patients (n = 36) were recruited in this study. Fresh blood of patients was collected before and after ART; peripheral blood mononuclear cells were isolated and stored at liquid nitrogen. Flow cytometry was performed as described in methods and materials. Frequency (A) and count (B) of DN T cells are compared among three groups. Mann–Whitney U test was used for statistical analysis. Horizontal lines indicate mean values, and error bars represent mean ± SEM (***p < 0.0001). [file Image_1.TIF]
